# Imaging Photoelectron Circular Dichroism in the Detachment of Mass‐Selected Chiral Anions

**DOI:** 10.1002/anie.202212020

**Published:** 2022-11-30

**Authors:** Jenny Triptow, André Fielicke, Gerard Meijer, Mallory Green

**Affiliations:** ^1^ Fritz-Haber-Institut der Max-Planck-Gesellschaft Faradayweg 4–6 14195 Berlin Germany

**Keywords:** Anions, Circular Dichroism, Mass Selection, Photoelectron Spectroscopy, Velocity Map Imaging

## Abstract

Photoelectron Circular Dichroism (PECD) is a forward‐backward asymmetry in the photoemission from a non‐racemic sample induced by circularly polarized light. PECD spectroscopy has potential analytical advantages for chiral discrimination over other chiroptical methods due to its increased sensitivity to the chiral potential of the molecule. The use of anions for PECD spectroscopy allows for mass‐selectivity and provides a path to simple experimental schemes that employ table‐top light sources. Evidence of PECD for anions is limited, and insight into the forces that govern PECD electron dynamics in photodetachment is absent. Here, we demonstrate a PECD effect in the photodetachment of mass‐selected deprotonated 1‐indanol anions. By utilizing velocity map imaging photoelectron spectroscopy with a tunable light source, we determine the energy‐resolved PECD over a wide range of photon energies. The observed PECD reaches up to 11 %, similar to what has been measured for neutral species.

## Graphical abstract

Energy‐resolved Photoelectron Circular Dichroism (PECD) is demonstrated in the photoelectron spectroscopy of mass‐selected deprotonated 1‐indanol anionic ([Ind‐H]^−^) enantiomers. A unique PECD signature is observed for several isomers and detachment channels of [Ind‐H]^−^. This work supports the use of anions for future analytical methods of enantiomeric discrimination, and allows for insight into the influence of short‐range interactions on PECD.

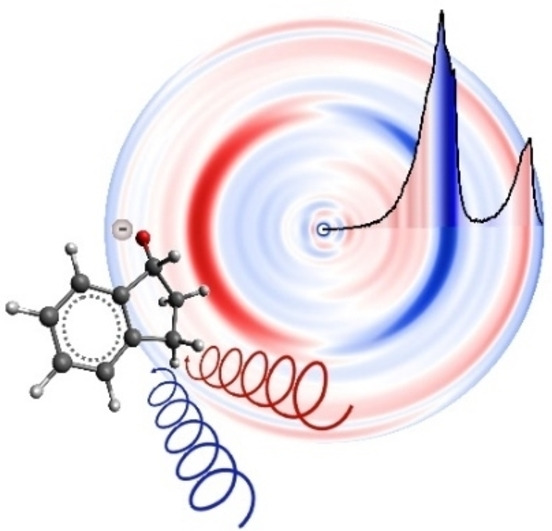

Determination of the enantiomeric excess of a mixed solution of chiral molecules has been pursued by a broad scientific community for many years. Conventional chiroptical methods (such as absorption circular dichroism, Raman optical activity, or polarimetry) rely on interactions with a molecule's magnetic moment to invoke a chiral response. These interactions are weak, thus requiring significantly concentrated samples in solution for analysis. Recently, a new generation of chiroptical techniques has emerged, whose sensitivity to molecular chirality can be rationalized within the electric dipole approximation. As a result, these techniques produce chiral signals that are orders of magnitude greater compared to the methods mentioned above, enabling chiral analysis to be effectively conducted in the gas‐phase. This class of techniques includes enantiomer specific microwave spectroscopy (ESMS),^[1]^ Coulomb explosion imaging^[2]^ and photoelectron circular dichroism (PECD).^[3]^ Here, we single out PECD for its analytical potential for analysis of dilute, multi‐component chiral samples, and interesting electron dynamics.

Irradiation of a non‐racemic sample by circularly polarized light (CPL), which results in the removal of an electron from the chiral molecule, leads to the chiral signature of PECD. This signature is a forward‐backward (FW/BW) asymmetry in the photoelectron angular distribution, with respect to the axis of photon propagation. The asymmetry in the electron flux is inverted for the other enantiomer, or when the excitation is conducted using the opposite circular polarization of light. The PECD signal for a given enantiomer is defined as:^1^

(1) $$PECD\left(\theta ,r\right)=2\frac{{I(\theta ,r)}_{LCP}-{I(\theta ,r)}_{RCP}}{{I(\theta ,r)}_{LCP}+{I(\theta ,r)}_{RCP}}$$

where *I*(*θ*,*r*)_(LCP/RCP)_ are radial electron yields in a given direction. The difference between detachment by left circular polarized light (LCP) and right circular polarized light (RCP) is normalized to the total electron yield.^[4]^

PECD was first predicted in 1976,^[5]^ and experimental confirmation of this effect arrived in the early 2000’s with the advent of circularly polarized synchrotron light sources.[^3b^, ^4^, ^6^] These light sources enabled the efficient single‐photon ionization of neutral gas‐phase molecules by either VUV or X‐ray radiation. A particular success for investigating angle‐ and energy‐resolved photoionization PECD resulted from the combination with velocity map imaging (VMI) photoelectron spectroscopy (PES). PES is a well‐known spectroscopic method with lenient transition rules, thereby making it a versatile technique suitable for investigations into a variety of unique chemical systems.^[7]^ VMI has been combined with PES (VMI‐PES) to enable the acquisition of the full angular distribution of the photoelectron flux at near‐100 % collection efficiencies.^[8]^ The VMI‐PES combination has been used in the study of valence‐shell PECD for a variety of chiral compounds,^[9]^ to provide a growing understanding of the PECD signal's sensitivity to ionization channel, vibrational population,^[10]^ isomerism,^[11]^ conformation,^[12]^ and condensation effects.^[13]^ As the variety of chiral species that are characterized by a sizable PECD signal grows, this phenomenon continues to make a case for itself as a potentially versatile analytical technique for determining enantiomeric excess.

In order to realize the analytical potential of this technique, an approach that provides solid information about the molecular source of the photoelectrons, and replaces the use of synchrotron light sources with table‐top lasers would be ideal. Photoelectron photoion coincidence (PEPICO) spectroscopy has been previously employed in order to provide a mass‐tagged PECD signal.[^11^, ^14^] PECD measurements have even been conducted in coincidence with photoion circular dichroism (PICD), which determines the asymmetry in ion counts from ionization by LCP and RCP radiation.^[15]^ This type of coincidence study enables two dichroism measurements for a single identified mass. However, as PEPICO experiments enable mass determination post ionization, they can be hindered by multiple ionization products. Increasingly complex samples may become too challenging for these methods.

A transition from synchrotron light sources to table‐top lasers has been managed with multiphoton PECD (MP‐PECD), utilizing both femtosecond[^14^, ^16^] and nanosecond lasers.^[17]^ Such ionization schemes have been implemented with PEPICO spectroscopy to provide a mass‐selective table‐top experiment. Furthermore, resonantly enhanced multiphoton ionization (REMPI) provides a conformer‐selective scheme for PECD.[^16b^, ^17^] However, electron scattering dynamics are inherently more complicated in these ionization schemes, and uncertainty in the effects of the intermediate state can make the final PECD assignments ambiguous.[^9^, ^19^]

The investigation of anions utilizing PECD spectroscopy has both analytical benefits and opportunities for furthering understanding of electron dynamics of this effect. In an analytical context, anions allow for mass selection before photodetachment, thereby simplifying final assignments. Also, the lower electron detachment energies, in comparison to neutral molecules, enable single‐photon electron detachment by ns light pulses from more common UV/Vis lasers. Such analytical advantages have been demonstrated in the electron yield CD of DNA polyanions.^[20]^ From a fundamental perspective, the study of anions provides additional insight into the effects of short‐range interactions on the PECD signal, as the long‐range interactions between a departing electron and the parent molecule can in most cases assumed to be negligible. Despite these advantages of studying the PECD of anions, there have been very few studies on this topic.

To date, there are only two published experimental studies^[21]^ and one theoretical study^[22]^ reporting on the PECD effect in anions. The experimental work demonstrated an electron asymmetry for two amino acid anions and for the peptide gramicidin, using a split detector to count the photoelectrons in the forward and backward direction. A PECD signal of up to 4 % has been reported in these anions.^[21a]^

We have built on the success of current experimental work by coupling the PECD technique with pre‐photodetachment mass selection, with VMI‐PES, and with a tunable light source to provide an energy‐resolved PECD signal for the isolated deprotonated 1‐indanol anion ([Ind‐H]^−^). Indanol was selected for its rigidity in order to reduce the number of conformers possibly contributing to the photoelectron spectrum. Also, the recent work on the PECD of neutral 1‐indanol enables the direct comparison of similar detachment channels between the deprotonated anion and closed shell neutral.^[12c]^

Using a plasma entrainment source,^[23]^ [Ind‐H]^−^ anions are produced by intersecting an expansion containing OH^−^ with an Ar expansion containing vaporized 1‐indanol. The resulting anionic beam is mass separated by a time‐of‐flight mass spectrometer, (an example mass spectrum is provided in the Supporting Information, Figure S1). This separation ensures that only anions of a single mass are present in the detachment region at the time of electron detachment. Selected anions are intercepted by the polarized output of an OPO pumped by a ns Nd:YAG laser. Photodetached electrons are focused onto a position sensitive detector by velocity map imaging optics.^[8]^ By this method, we are able to recover the full photoelectron angular distribution of the anion.

Photoelectron spectra of [Ind‐H]^−^ (Figure 1) have been acquired for a wide selection of photon energies (*hν*), ranging from 2.34 eV to 4.96 eV. The spectra are obtained from the distributions of radial projections of electrons with unique kinetic energies, which carry angular information with respect to the polarization or propagation axis of the light (Figure 2). These distributions have been analyzed using polar onion peeling (POP), which reconstructs the full radial 2D‐electron projection into a linear photoelectron spectrum, and provides the angular information of the detachment channels.^[24]^ In Figure 1, the spectrum taken at *hν*=4.86 eV shows three main features, labeled A, B, and C. The maxima of these peaks correspond to the detachment energies of about 1.1 eV, 2.4 eV, and 4.6 eV, respectively. These features have been assigned through comparisons with DFT/B3LYP‐D3/aug‐cc‐pVTZ calculations. Features B and C are attributed to detachment from the three highest occupied orbitals of the lowest energy deprotonated tautomer, where the deprotonation occurs at the hydroxy group ([Ind(O)‐H]^−^). The formation of this tautomer is calculated to be exothermic, resulting in an excess energy of 0.90 eV. Although other works have shown a single conformer of neutral 1‐indanol present in an expansion of argon, this excess energy is expected to enable the formation of additional isomers.[^12c^, ^25^]

**Figure 1:**
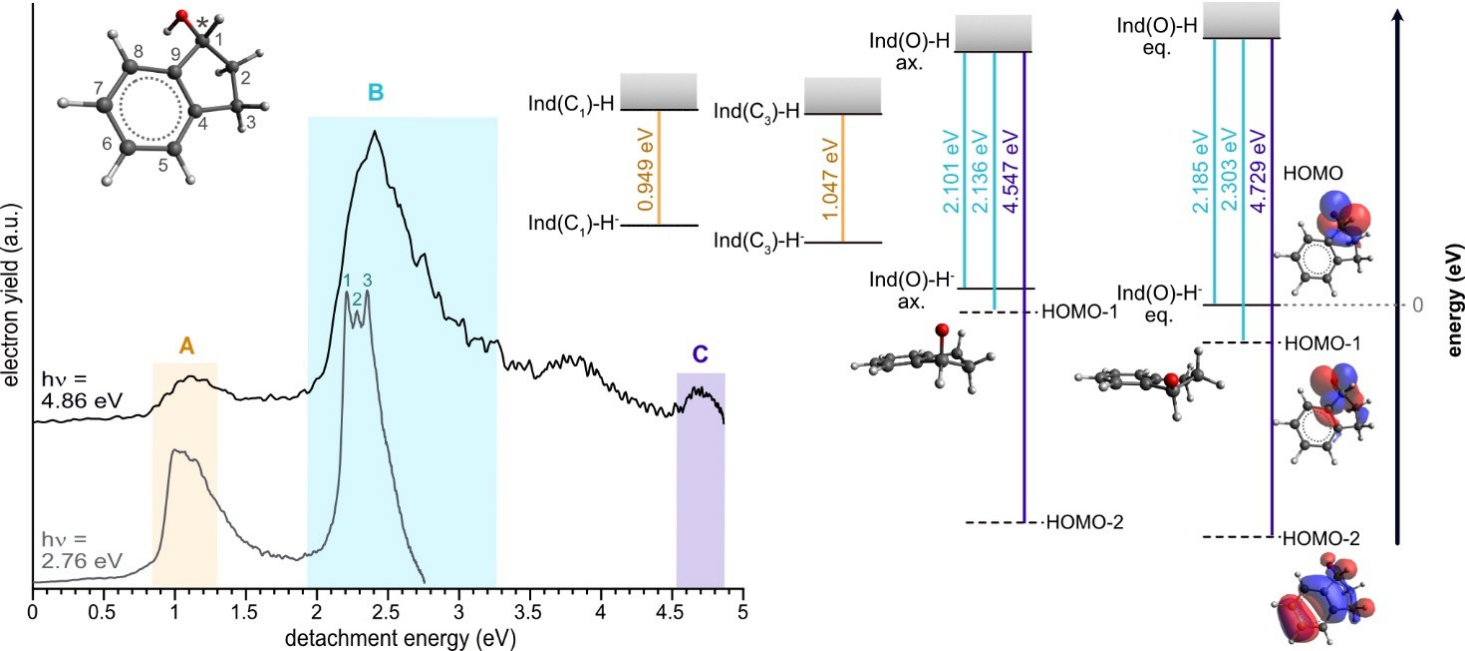
Photoelectron spectra of [Ind‐H]^−^ and calculated detachment energies for expected channels of photodetachment. Top Left: (R)‐1‐indanol with carbon atoms numbered to indicate deprotonation site. The stereogenic carbon atom is marked by an asterisk. Left: Two photoelectron spectra that have been acquired at two different photon energies (top: 4.86 eV, bottom: 2.76 eV). Photoelectron spectra are plotted in detachment energy and electron yield, where the baselines of the spectra are offset vertically to provide visual clarity. The features in the top spectrum are labelled alphabetically (A, B, C). The lower spectrum indicates higher resolution sub‐features (B_1_, B_2_, B_3_), which contribute to feature B. Spectral regions are color‐coded by the predicted transitions (right) assigned to them. Right: Energy level diagram demonstrating the predicted transitions of the two conformers (eq, ax) of the ground state tautomer ([Ind(O)‐H]^−^) and the two higher energy tautomers, [Ind(C_3_)‐H]^−^ and [Ind(C_1_)‐H]^−^. Anion to neutral ground state (solid black lines) transitions are calculated vertical detachment energies. Detachment energies out of the lower lying orbitals (dotted lines) of the [Ind(O)‐H]^−^ conformers have been calculated using Koopmans’ theorem.^[18]^ All displayed energy levels are referenced to the ground state of the lowest energy conformer of [Ind(O)‐H]^−^ (*E*=0 eV).

**Figure 2:**
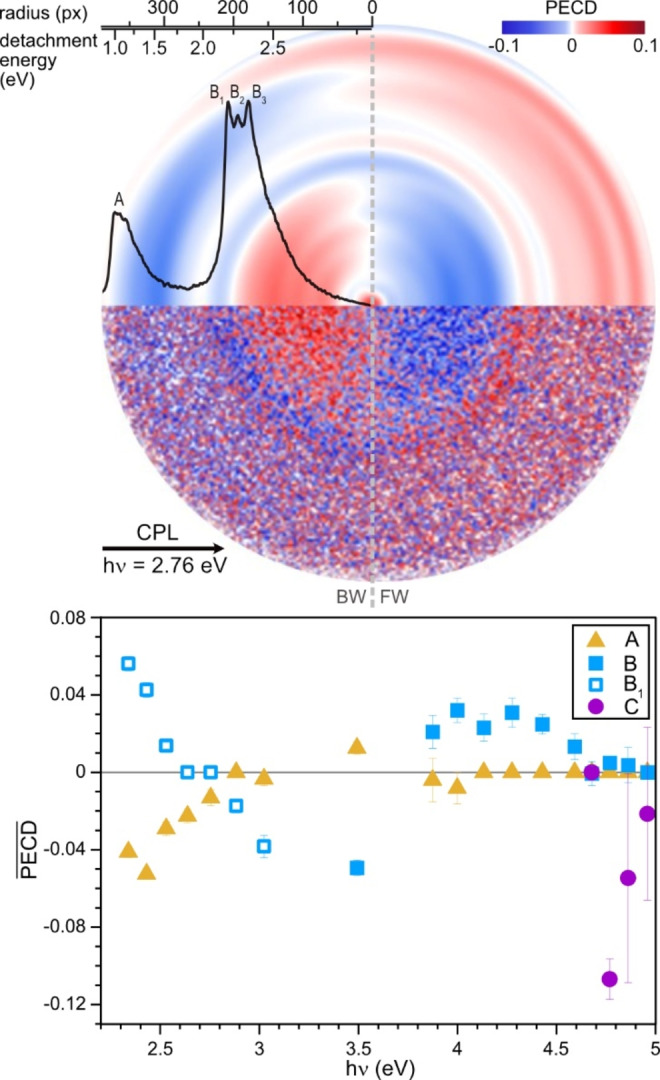
Top: Raw and reconstructed PECD angular distributions, taken at *hν*=2.76 eV. The black arrow indicates the propagation direction of light. The reconstructed spectrum is scaled for radial distance and detachment energy. Bottom: Mean PECD of the 3 main spectral features plotted across photon energy (*hν*). For the *hν* range 2.34–3.02 eV, band B is represented by the sub band, B_1_.

For [Ind(O)‐H]^−^, two conformers have been predicted, where the inversion of the aliphatic ring leads to either an equatorial ([Ind(O)‐H]^−^ eq) or axial ([Ind(O)‐H]^−^ ax) position of the alkoxide, with respect to the 5‐membered ring. [Ind(O)‐H]^−^ eq is the lowest energy conformer and has a calculated vertical detachment energy (VDE) of 2.185 eV. The axial conformer is predicted to lie only 0.069 eV above the equatorial conformer and has a similar VDE of 2.101 eV. These predicted values are close to the detachment energy of feature B, indicating the possibility of both conformers contributing to this feature. The assignment of feature B is additionally complicated by the possibility of detachment from the energetically close HOMO‐1 orbital. The possibility of detachment from the HOMO‐1 orbitals of the conformers is supported by the assignment of feature C to detachment from the HOMO‐2 orbital of [Ind(O)‐H]^−^.

Feature A can be attributed to a combination of two higher energy tautomers, where deprotonation occurs at the C_3_ position ([Ind(C_3_)‐H]^−^), or the C_1_ position ([Ind(C_1_)‐H]^−^). It is important to note that C_1_ is the stereogenic center of 1‐indanol, and deprotonation at this position leads to an achiral tautomer. These tautomers are expected to have a similar conformational landscape to [Ind(O)‐H]^−^. Other tautomers are not expected as their acidities are too low for deprotonation by OH^−^ (see Table S1). It is clear from this assignment, that regardless of single mass analysis and careful molecular selection, the formation of anions by deprotonation in the gas phase can still leave one with complex mixtures of isomers.

An advantage of coupling VMI detection with a tunable laser is the ability to reach higher absolute electron kinetic energy (eKE) resolution when utilizing *hν* near the threshold of detachment.^[26]^ Such an improvement in resolution can allow for a more detailed assignment of the spectrum. In the lower spectrum of Figure 1 measured at *hν*=2.76 eV, the broad feature B seen at *hν*=4.86 eV reveals at least three, reproducible, narrower features (B_1_, B_2_, B_3_). These peaks have a center detachment energy of 2.21(1) eV, 2.28(1) eV, and 2.35(1) eV. As is indicated above, feature B is expected to be comprised of four electronic transitions. Comparisons between the calculated detachment energies of these four transitions and the higher resolution spectrum do not immediately reveal a unique assignment. The spacing between peaks B_1_ and B_3_ (0.14 eV) is similar to the frequency calculated for the CO‐stretch of the neutral radical (1092 cm^−1^, 0.135 eV). A Franck–Condon simulation of the [Ind(O)‐H]^−^ eq HOMO detachment supports such assignment (see Figure S2). A similar simulation has been conducted for the axial conformer, but it's potential contribution to the spectrum is unclear. The expected accuracy of the calculations and the current experimental resolution do not allow for a confident assignment of peak B_2_. However, the agreement between our experimental results and the FC simulation suggests that partial vibrational resolution is observed.

It has been explicitly verified that the relative change in eKE for a given transition (A, B, C) matches that of a single photon process (Figure S3). Therefore, we conclude that the observed PECD originates from the electron detachment of anions; potential contributions resulting from detachment followed by multiphoton ionization of neutral radicals are negligible, or entirely absent.

Quantitative PECD values are determined through a polar onion peeling procedure of the LCP and RCP images, as described in the Supporting Information. Electron angular distributions, reconstructed using rBasex, are provided as a visual aid.^[27]^ The reconstructed images allow for the more subtle PECD signals to be visualized, as is evident in the comparison of the raw and reconstructed PECD images displayed in Figure 2. These images demonstrate a reversal in signal across the central axis, defining the separation of the FW and BW halves of the image. The imperfections in anti‐symmetry across the central axis of the reconstructed image can be caused by slight deformations in the circularity of the raw images or by non‐uniform sensitivity across the detector. It is clear from the alternation of PECD evident in a single half of the image that each transition possesses a unique PECD signature. Close evaluation of the PECD for the B_1_, B_2_, and B_3_ signals reveals that even these narrower features possess distinct PECD. This is evident from the color change from blue via white to red in the reconstructed image over the corresponding energy range (see Figure 2 and S4).

The PECD for each feature has been analyzed for the full range of *hν* sampled. In Figure 2, a plot of the PECD for features A, B and C against *hν* reveals the PECD to be eKE dependent for each feature, which is consistent with what has been established for neutral species.[^3b^, ^4^, ^6^] The largest mean PECD observed is for feature C with a value of −0.11, or a PECD of 11 %. It is interesting to see a substantial PECD for feature A, considering some anticipated contributions to the electron yield by the detachment of the achiral tautomer. Additionally, significant PECD for features A and B are evident at high hν, which correspond to eKEs greater than 1 eV. The wavefunction of the departing electron in the photodetachment of an anion may be described as a plane wave.^[28]^ PECD has been predicted to be vanishing for plane wave systems.^[29]^ Such a description would suggest that a PECD signal would only be observable in anions for very low eKEs, where the plane wavefunction description does not hold. However, this is not what we observe. The recent theoretical work investigating the PECD of anions does emphasize the importance of short‐range interactions in the observation of a PECD signal, but it does indicate that a PECD could be measured for photodetached electrons carrying up to 12 eV of kinetic energy.^[22]^ As detachment of electrons from anions is the reverse process of electron attachment to a neutral molecule, insight into the universal electron dynamics that govern the PECD effect can likely be gained from those studies as well.^[30]^

As we have measured the PECD for a wide range of *hν*, it is possible to compare analogous detachment channels in the anion and neutral molecule at similar eKEs. The HOMO and HOMO‐1 orbitals of [Ind(O)‐H]^−^ (feature B) are mainly nonbonding, lone‐pair, oxygen‐centered orbitals, similar to the HOMO‐2 orbital of neutral 1‐indanol.^[12c]^ Detachment out of these alike orbitals have been carried out at eKEs of 0.65 eV and 1.7 eV for both species, albeit for opposite enantiomers.^[12c]^ At eKE ≈1.7 eV the PECD of the anion is about 4 %, whereas the PECD for the neutral molecule is about 6 %. At eKE ≈0.65 eV, the magnitudes of PECD in the anion and neutral molecule are both ≈4 %. However, moving to lower energies, we observe a sign flip in the anion, which is absent in 1‐indanol. The similarity in magnitude of the PECD signal of 1‐indanol and [Ind‐H]^−^ suggests that long‐range interactions might not be needed to produce a sufficient PECD signal.

Not only can a difference in electron angular flux be observed upon switching the polarization of light, but basic symmetry considerations require a reversal of the FW‐BW asymmetry with exchange of enantiomer. In Figure 3 we show the PECD observed for the R and S enantiomers, and the racemic mixture of [Ind‐H]^−^ at three *hν*. For each *hν*, one feature has been selected to demonstrate the reversal in the sign of the PECD between the enantiomers. Full reconstructed distributions and corresponding raw distributions at each *hν* are found in the Supporting Information (Figure S5). The racemic sample produces no asymmetry, which verifies that our observed asymmetry cannot be an effect of inherent asymmetries of our instrument. For features B and C, the magnitude of the PECD is the same within the reported experimental error. Although feature A does show a sign reversal for the asymmetry, the magnitude of PECD in the S‐enantiomer is slightly smaller than the magnitude of PECD in the R‐enantiomer. This discrepancy might arise from the slightly different source conditions between the two experiments, leading to different contributions of the chiral and achiral tautomers to this spectral feature. It is noted that the error bars are solely derived from the deconvolution procedure of the images and do not include considerations of the purity of the laser polarization or starting compound.

**Figure 3:**
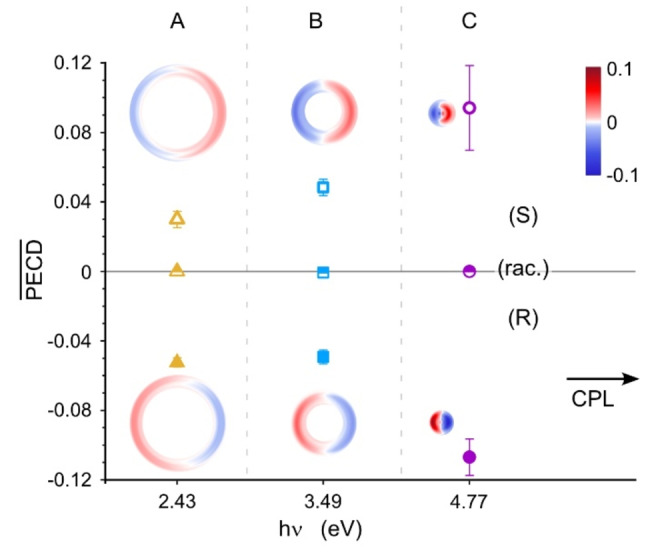
Comparison of the mean PECD for features A, B, and C of the two enantiomers and the racemic mixture. The angular distributions have been partially masked in order to highlight the corresponding band in the graph below. For each selected photon energy, one feature is shown. Full reconstructed electron distributions are provided in Figure S5.

By utilizing VMI‐PES, in combination with mass spectrometry and tunable photodetachment, we have demonstrated an energy‐resolved PECD signal for the mass selected, deprotonated (R)‐ and (S)‐1‐indanol anion over a wide range of photon energies. It is clear from our photoelectron spectrum that many isomers can contribute to the photoelectron signal, each providing a unique PECD signature. As PECD is known to be sensitive to conformation and isomerism, the coupling of PECD with a spectroscopic method is crucial in systematically working towards an analytical PECD technique. In addition, an ion source with softer ionization conditions could act to limit convolution of the spectrum by reducing the number of isomers formed in the molecular beam. Weitzel et al. have previously demonstrated the utility of electrospray ionization, a well‐known soft ionization method, in the formation of anions for PECD studies.^[21]^ We expect the coupling of an ESI source with pre‐photodetachment mass selection and VMI‐PES would be well‐suited for single compound enantiomer‐specific information within complex, multi‐component samples.

By providing comparisons to current theoretical predictions and to the experimental results for neutral 1‐indanol, we have taken the first steps in exploring the role of short‐range interactions in PECD. With the observation of a [Ind‐H]^−^ PECD that is similar in magnitude to that of neutral 1‐indanol, it is clear that well accepted descriptions of the electron dynamics, which govern the PECD effect, fail to describe the specific dynamics that arise in photodetachment of anions.

## Conflict of interest

There is no conflict of interest to report.

## Supporting information

As a service to our authors and readers, this journal provides supporting information supplied by the authors. Such materials are peer reviewed and may be re‐organized for online delivery, but are not copy‐edited or typeset. Technical support issues arising from supporting information (other than missing files) should be addressed to the authors.

Supporting Information

## Data Availability

The data that support the findings of this study are available in the Supporting Information.

## Footnotes

n1 This equation is consistent with the one used in the references herein, with the exception of reference [21], whose definition results in a factor two larger PECD effect.
