# Supplementary material for: Imaging Photoelectron Circular Dichroism in the Detachment of Mass‐Selected Chiral Anions
Source: Angew Chem Int Ed Engl. 2022 Nov 30;62(1):e202212020. doi: 10.1002/anie.202212020 (PMC10099851; doi:10.1002/anie.202212020)
Supplement: Supplementary file 1 — Supporting Information [file ANIE-62-0-s001.pdf]

Angewandte Chemie
Eine Zeitschrift der Gesellschaft Deutscher Chemiker

Supporting Information

Imaging Photoelectron Circular Dichroism in the Detachment of Mass-Selected Chiral Anions

*J. Triptow, A. Fielicke, G. Meijer, M. Green**

Table of Contents

Table of Contents 2
Experimental Procedures 2
VMI Spectroscopy 2
PECD 2
Computational Methods 3
Results and Discussion 3
Mass Spectrum 3
Tautomer Relative Energies 4
Franck-Condon Simulations of the Vibronic Structure of Feature B 4
Confirmation of Single-Photon Electron Detachment 5
PECD Analysis of the B_1, B_2 and B_3 Peaks 5
Full Reconstructed Electron Angular Distributions and Raw PECD Images 6
References 6
Author Contributions 6

Experimental Procedures

VMI Spectroscopy

$[Ind\text{-}H]^-$ anions are investigated using a home-built instrument consisting of a plasma entrainment source, a time-of-flight mass spectrometer, and a velocity map imaging (VMI) spectrometer. The mass spectrometer and VMI spectrometer have been described in detail elsewhere.[1] The anionic source is a replica of the pulsed anionic plasma entrainment source by Lineberger et al.[2] $[Ind\text{-}H]^-$ is formed by heating either (R)-(-)-1-indanol or (S)-(+)-1-indanol placed behind a pulsed valve to 373 K, which is then seeded into a flow of argon gas and supersonically expanded into the vacuum. A second perpendicularly oriented pulsed valve coupled with a high voltage discharge produces a plasma containing OH^-, which is crossed with the indanol expansion. The OH^- reacts with the neutral indanol, leading to deprotonation, resulting in the formation of the anion. The anions are mass-separated via a Wiley McLaren time-of-flight mass spectrometer (WM-TOFMS).[3] The mass-selected anion is photodetached using an optical parametric oscillator (OPO Panther Ex, Continuum) system pumped by the third harmonic (355 nm) of a Surelite II Nd:YAG laser, at a photon energy, ranging from 2.34 to 4.96 eV. The detached photoelectrons are focused using Eppink and Parker VMI optics[4] onto a detector, which is comprised of a set of two microchannel plates in a chevron orientation, coupled to a P43 phosphor screen. The incident electrons are recorded using a CCD camera. Photoelectron events are accumulated into a photoelectron image. The VMI electron spectrometer is calibrated with S^-, using the precisely known anion to neutral transitions. The VMI spectrometer can achieve an electron kinetic energy (eKE) resolution of ΔeKE/eKE = 3.4 %. The instrument runs at a 10 Hz frequency, where photoelectron angular distributions with a count rate of 5-50 electrons/cycle are accumulated for a total of 10^6-10^7 electron counts. The electron distributions are re-constructed using the polar onion peeling method (POP) to provide a final photoelectron spectrum. Reconstructed PECD photoelectron distributions are produced using rbasex in Pyabel package, and a low pass filter is applied to remove artificial frequencies generated by the inversion.

PECD

To measure the PECD at a given $h\nu$, electron angular distributions are acquired using LCP and RCP irradiation, which is switched shot-to-shot with a photoelastic modulator (PEM) that acts as a rapidly adjustable $\lambda/4$ plate. The different distributions generated by LCP and RCP radiation are binned subsequently in order to minimize the possibility of an artificial PECD effect being measured due to changes in signal over time. The asymmetric distributions are then analyzed by applying polar onion peeling to the forward (FW) and backward (BW) hemispheres of each polarization image separately. A PECD for each hemisphere is determined by:

$$PECD_H = 2 * \frac{I_{LCP,H} - I_{RCP,H}}{I_{LCP,H} + I_{RCP,H}} \quad \text{where} \quad H = FW \ or \ BW$$

The mean PECD ($\overline{PECD}$) is the average of the PECD determined in the forward direction and in the backward direction:

$$\overline{PECD} = \frac{PECD_{FW} - PECD_{BW}}{2}$$

Reconstructed PECD images are obtained by applying rBasex, implemented through PyAbel, to the raw PECD image, followed by a low pass filter to remove artifacts generated by the inversion. The reconstructed images are an aid for visualizing the PECD asymmetry, they are not used to provide quantitative information.

Computational Methods

DFT calculations of the $[Ind\text{-}H]^-$ tautomers and conformers have been carried out with the hybrid density functional B3LYP[5] and the D3 dispersion correction of Grimme (B3LYP-D3).[6] The augmented correlation-consistent polarized valence-only triple-zeta basis set (aug-cc-pVTZ) is used,[7] and all calculations are performed with the GAUSSIAN16 software package.[8] The optimized geometries, molecular energies, frequencies and molecular orbitals have been calculated for all potential deprotonated tautomers. For the lowest energy tautomer, $[Ind(O)\text{-}H]^-$, the two conformers have been optimized. Vertical detachment energies (VDEs) have been computed by also including energy calculations of the neutral molecules in the anion ground state geometry. To calculate the detachment energy out of lower energy orbitals, Koopmans' theorem is used.

Results and Discussion

Mass Spectrum

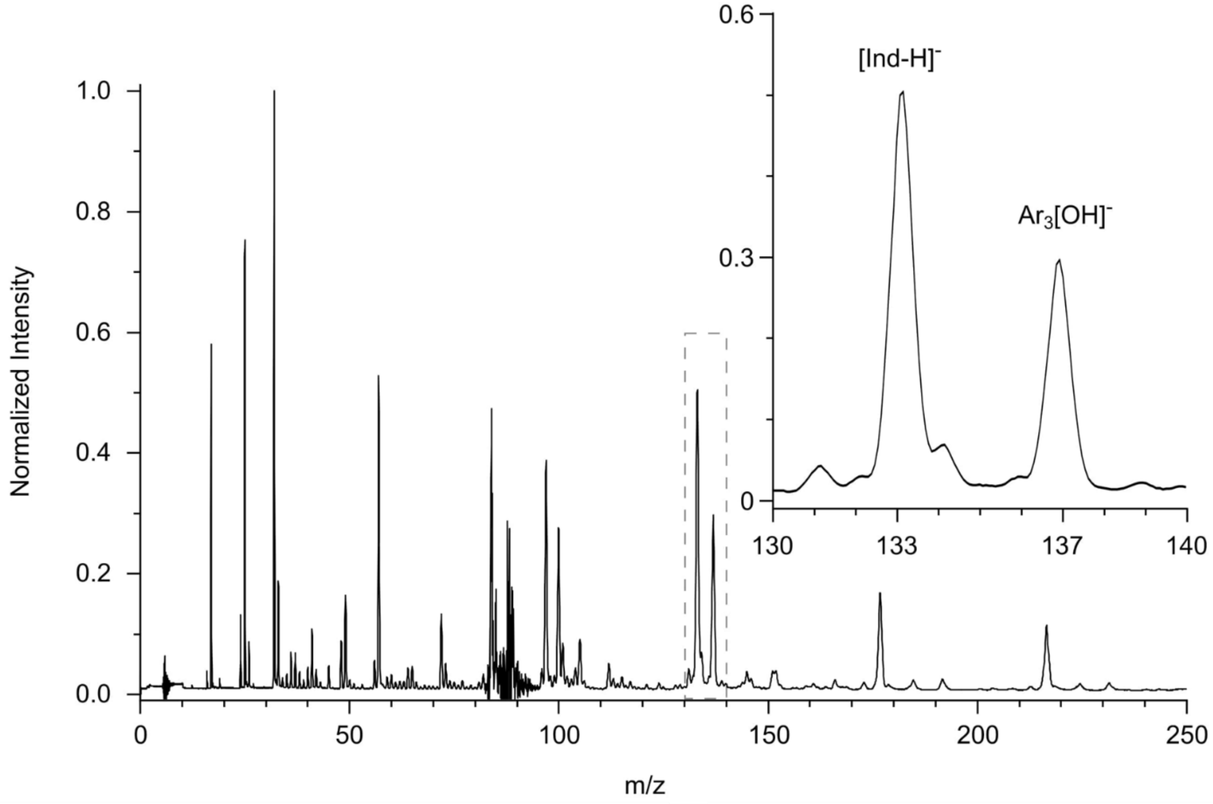

Figure S1. Mass spectrum of the anions generated through plasma entrainment of an argon expansion seeded with indanol. The mass spectrum includes the target anion ($[Ind\text{-}H]^-$), as well as ions generated in the plasma formation (OH^-, O^-), S^- ions used for calibration, and argon clusters of the smaller ions. Fluctuations at approximately m/z ~ 5 and 85 are attributed to noise caused by HV switches. The inset (top right) highlights our anion of interest.

Tautomer Relative Energies

Table S1. Calculated relative energies and vertical detachment energies of the deprotonated $[Ind-H]^{-}$ tautomers.

Tautomer	Relative Energy (eV)	Vertical Detachment Energy (eV)
$[Ind(O)-H]^{-}$	0	2.042
$[Ind(C_1)-H]^{-}$	0.603	0.949
$[Ind(C_2)-H]^{-}$	1.001	1.122
$[Ind(C_3)-H]^{-}$	0.466	1.047
$[Ind(C_5)-H]^{-}$	1.214	1.704
$[Ind(C_6)-H]^{-}$	1.322	1.617
$[Ind(C_7)-H]^{-}$	1.329	1.628
$[Ind(C_8)-H]^{-}$	0.966	2.110

Franck-Condon Simulations of the Vibronic Structure of Feature B

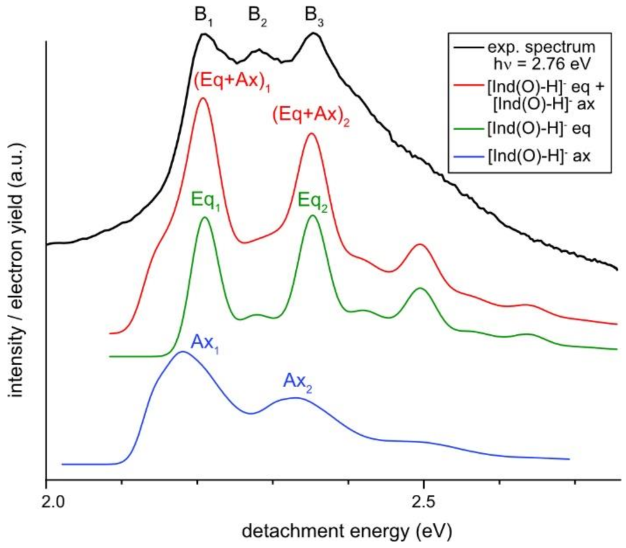

Figure S2. Comparison of the simulated vibronic spectra of $[Ind(O)-H]^{-}$ eq and $[Ind(O)-H]^{-}$ ax with the experimental spectrum taken at hν = 2.76 eV. The combination simulation (Eq + Ax) assumes a 1:1 ratio of the conformers.

Franck-Condon simulations have been performed using Gaussian 16 for the two conformers of $[Ind(O)-H]^{-}$, with a simulation temperature of 0 K. The unscaled frequencies of the optimized geometries have been used in the simulation, and the origins of the predicted vibronic spectra have been shifted to the origin of the experimental spectral band. A Gaussian distribution of HWHM = 160 cm^{-1} (0.02 eV) has been applied in order to produce simulations with similar spectral resolution to the experimental spectrum taken at hν = 2.76 eV. The predicted vibronic spectra of the eq and ax conformers of $[Ind(O)-H]^{-}$ each show two intense features in the energy range of the B_1, B_2, B_3 peaks. The center of each first peak ($(Eq+Ax)_1$, Eq_1, and Ax_1) corresponds to the origin of transition. The second most prominent peak at higher detachment energy ($(Eq+Ax)_2$, Eq_2, and Ax_2) is dominated by vibrational modes involving a C-O stretch activation. The eq conformer has an energy spacing very similar to the difference in energy between the B_1 and B_3 peaks, and the broadening profile is quite similar. The ax conformer shows significant contributions from other vibrations, leading to bands that are much broader than that which is shown for the experimental spectrum, and the relative energy difference is less similar. As the eq and ax conformers are very close in energy, it also is possible for there to be an even distribution of these conformers in the measurement. The simulated combination spectrum looks to still be quite dominated by the transitions of the eq conformer. Therefore, it is not clear what role the ax conformer plays in the experimental spectrum. Also, these simulations did not provide an assignment for the B_2 peak in our spectrum.

Confirmation of Single-Photon Electron Detachment

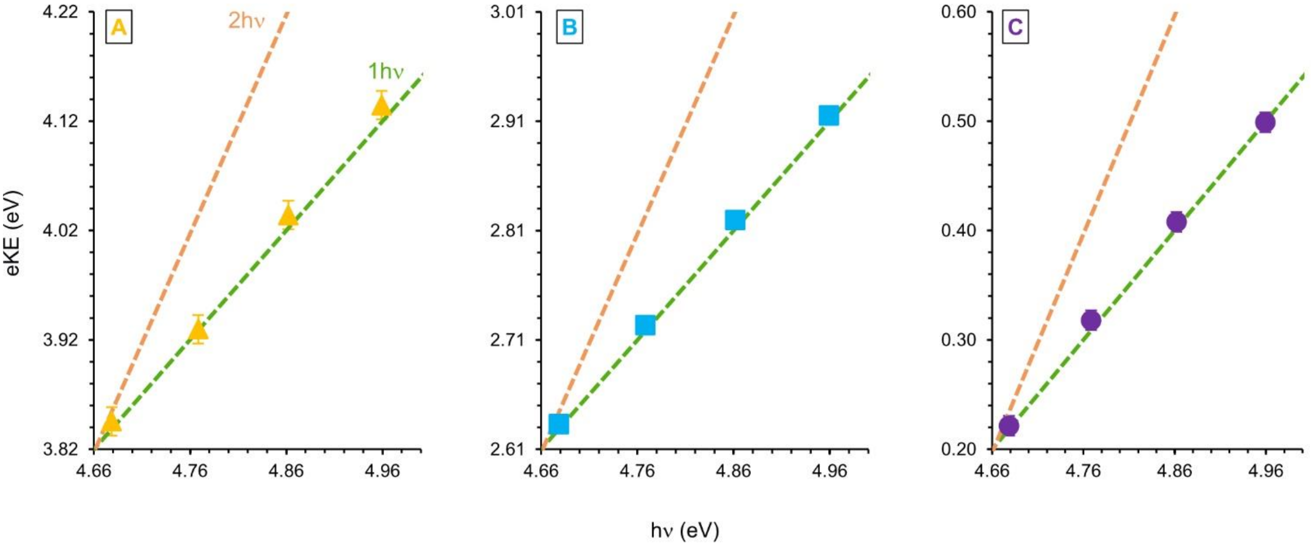

Figure S3. Plot of the low detachment energy threshold eKE of features A, B, and C against photon energy. Dashed lines indicate the expected eKEs for a one photon and two photon process.

PECD Analysis of the B_1, B_2 and B_3 Peaks

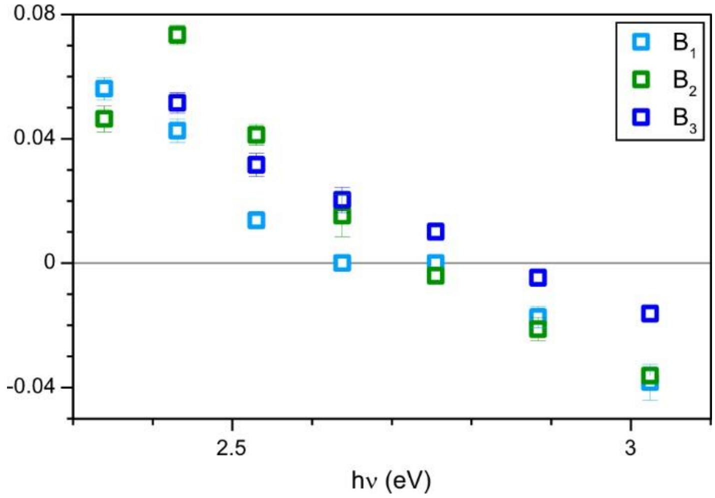

Figure S4. Mean PECD of B_1, B_2, and B_3, plotted across hν = 2.34 - 3.02 eV.

The higher energy resolution obtained by near-threshold detachment enables the analysis of PECD for the energetically-close peaks B_1, B_2, and B_3. As is shown in figure 2, the three peaks share a similar trend, but each peak possesses a unique photon energy dependence. For this high-resolution area, it is possible to even track the peak-specific change in PECD sign. As peaks B_1 and B_3 have, tentatively, been assigned to a vibrational progression of the CO stretch, this capability could lead to further understanding of the relationship between vibrational population and resulting PECD signal.

Full Reconstructed Electron Angular Distributions and Raw PECD Images

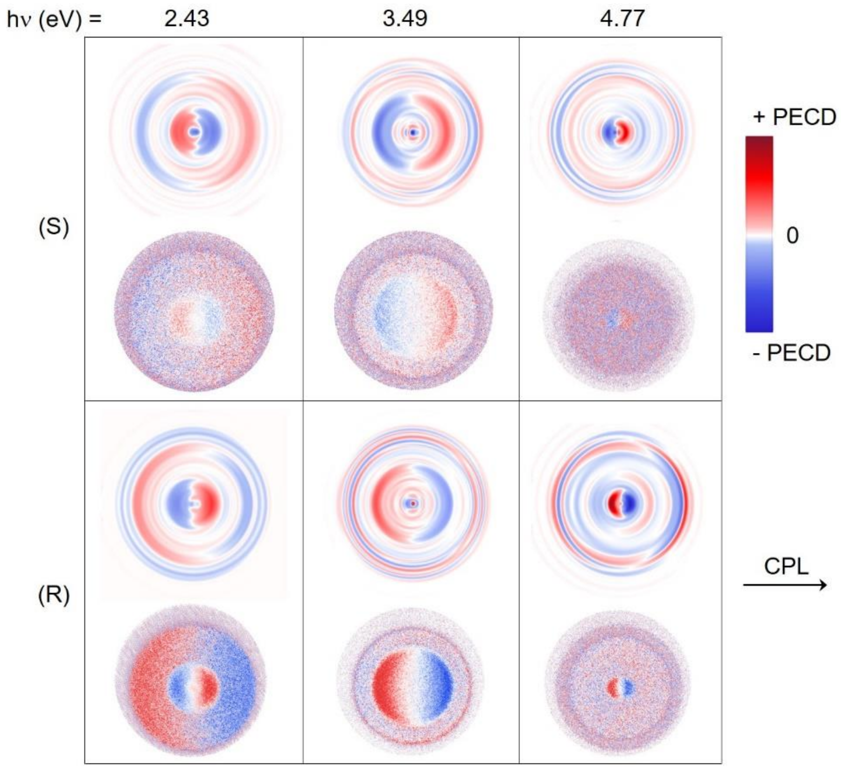

Figure S5. Full reconstructed electron angular distributions (top) and raw PECD images (bottom) for the (R) and (S) enantiomers of [Ind-H]$^{-}$, taken at hν = 2.43, 3.49, 4.77 eV. In the raw images the sharp outer ring indicates the edge of the detector.

References

[1] J. Triptow, G. Meijer, A. Fielicke, O. Dopfer, M. Green, *The Journal of Physical Chemistry A* **2022**.
[2] Y.J. Lu, J. H. Lehman, W. C. Lineberger, *J. Chem. Phys.* **2015**, *142*, 044201.
[3] W. Wiley, I. H. McLaren, *Rev. Sci. Instrum.* **1955**, *26*, 1150-1157.
[4] A. T. Eppink, D. H. Parker, *Rev. Sci. Instrum.* **1997**, *68*, 3477-3484.
[5] (a) A. D. Beck, *J. Chem. Phys* **1993**, *98*, 5648-5646; (b) A. D. Becke, *Phys. Rev. A* **1988**, *38*, 3098; (c) C. Lee, W. Yang, R. G. Parr, *Phys. Rev. B* **1988**, *37*, 785.
[6] S. Grimme, J. Antony, S. Ehrlich, H. Krieg, *J. Chem. Phys.* **2010**, *132*, 154104.
[7] (a) T. H. Dunning Jr, *J. Chem. Phys.* **1989**, *90*, 1007-1023; (b) D. Feller, *J. Comput. Chem.* **1996**, *17*, 1571-1586; (c) R. A. Kendall, T. H. Dunning Jr, R. J. Harrison, *J. Chem. Phys.* **1992**, *96*, 6796-6806; (d) B. P. Pritchard, D. Altarawy, B. Didier, T. D. Gibson, T. L. Windus, *J. Chem. Inf. Model.* **2019**, *59*, 4814-4820; (e) K. L. Schuchardt, B. T. Didier, T. Elsethagen, L. Sun, V. Gurumoorthi, J. Chase, J. Li, T. L. Windus, *J. Chem. Inf. Model* **2007**, *47*, 1045-1052.
[8] M. Frisch, G. Trucks, H. Schlegel, G. Scuseria, M. Robb, J. Cheeseman, G. Scalmani, V. Barone, G. Petersson, H. Nakatsuji, Gaussian, Inc. Wallingford, CT, **2016**.

Author Contributions

JT: data curation, formal analysis, investigation, writing of original draft (figure generation, review)

AF: Conceptualization, funding acquisition, investigation, writing - review & editing, project administration

GM: project administration, investigation, writing of original draft (review and editing)

MG: Funding acquisition, investigation, formal analysis, validation, writing of original draft [lead], project administration [lead]
